# hsa-miR-199b-3p suppresses osteosarcoma progression by targeting CCDC88A, inhibiting epithelial-to-mesenchymal transition, and Wnt/beta-catenin signaling pathway

**DOI:** 10.1038/s41598-023-39537-0

**Published:** 2023-08-02

**Authors:** Dongsheng Zhu, Han Qi, Hongqi Zhu

**Affiliations:** 1 Department of Pediatric Surgery, The First People’s Hospital of Lianyungang, 182 Tongguan North Road, Lianyungang, 222000 Jiangsu People’s Republic of China; 2 Department of Emergency Surgery, The Second People’s Hospital of Lianyungang, 41 Hailian East Road, Lianyungang, 222000 Jiangsu People’s Republic of China

**Keywords:** Cancer, Cell biology, Genetics, Molecular biology, Biomarkers, Oncology

## Abstract

The present study investigated microRNA (miR)-199b-3p expression in osteosarcoma (OS) and aimed to identify its potential mechanism of action contributing to the development of this disease. Firstly, miR-199b-3p and coiled-coil domain containing 88A (CCDC88A) expression data were evaluated from Gene Expression Profiling Interactive Analysis and Kaplan Meier plotter was used to assess the survival data. By analyzing the GSE65071 dataset from gene expression omnibus, it was found that miR-199b-3p was expressed at a low level. By using reverse transcription-quantitative PCR analysis in OS cells and tissues, CCDC88A was found to be expressed at a high level. Moreover, TargetScan predicted CCDC88A to be a downstream target of miR-199b-3p. Luciferase reporter assays were used to verify this prediction. In vitro overexpression of miR-199b-3p decreased the invasive and proliferative activity of OS cells. Mechanistic studies indicated that decreased miR-199b-3p resulted in increased expression of CCDC88A. Concomitantly, it impeded the Wnt/beta-catenin pathway and the epithelial-to-mesenchymal transition process. Overall, the results of the present study emphasized the pivotal role of the miR-199b-3p in the formation and progression of OS, suggesting that it could be used as a potential tumor biomarker.

## Introduction

Osteosarcoma (OS) is a disease that affects most frequently children and adolescents. It is considered to be one of the most universal primary malicious bone sarcomas with a poor prognosis^1^. Surgical resection of all tumor sites, radiation therapy, and chemotherapy are the three main therapies used for the treatment of patients with OS^2^. Despite significant efforts made in OS treatment, the 5-year survival rate remains between 50 and 80% and the survival rates remain unchanged since 1980^3,4^. Therefore, in order to increase patient survival rate and inhibit OS progression, the identification of novel therapeutic targets and their molecular mechanisms is urgently required.

MicroRNAs (miRs) are one type of non-coding small RNAs with an approximate length of 21–23 nucleotides. Certain miRs have been reported to contribute to OS progression and may be used in the diagnosis, prognosis, and treatment of this disease^5–7^. Some studies have also found that miR affects the metastasis and invasion of osteosarcoma^8^. For example, down-regulation of miR-34a can activate epithelial–mesenchymal transition (EMT) and promote the metastasis of osteosarcoma^9^. LINC00210 modulated the radiosensitivity of osteosarcoma cells via the miR-342-3p/GFRA1 axis, making LINC00210 a novel target for improving radiotherapy efficiency in osteosarcoma^10^. Upregulated expression of miR-664a could have an inhibitory effect on MEG3 gene expression and migration of osteosarcoma cells^11^. MiR-659-3p inhibited osteosarcoma tumor progression and lung metastasis by inhibiting SRPK1 expression and potentially downstream cell proliferation, and epithelial-to-mesenchymal transition genes^12^. The high expression of miR-183 can inhibit the Wnt/beta-catenin pathway and inhibit the metastasis of osteosarcoma^13^. Previous research studies have found that miR-199b-3p can affect proliferation, differentiation, and apoptosis in a variety of tumors^14,15^. However, the role of miR-199b-3p in OS has not been previously reported.

Coiled-coil domain containing 88A (CCDC88A) can bind to the actin cytoskeleton. Akt is a serine/threonine kinase, which is required for directional cell migration, ultimately leading to invasion and metastasis in cancer cells^16^. Several reports have suggested that CCDC88A is involved in tumor progression. Knockdown of CCDC88A expression suppresses human pancreatic, skin, and breast cancer cell migration and invasion, and significantly inhibits primary tumorigenesis in vivo^17–19^, suggesting that CCDC88A has a key role in cancer development.

The present study we first showed that miR-199b-3p had a tumor-suppressive function in OS by suppressing OS cell growth, migration, and invasion. Then, we characterized CCDC88A as a direct target of miR-199b-3p. MiR-199b-3p interacted with the 3′UTR of CCDC88A to inhibit the EMT and Wnt/beta-catenin signaling pathway to mediate its tumor-suppressive effect on the proliferation and invasion of OS cells. These findings may be useful for the development of possible clinical approaches for OS treatment.

## Materials and methods

### Bioinformatic analysis

The overall survival of the patients with OS and different levels of miR-199b-3p expression was analyzed by Kaplan Meier plotter (http://kmplot.com/analysis/). The CCDC88A expression data and prognostic information were obtained by the Gene Expression Profiling Interactive Analysis (GEPIA) database (http://gepia.cancer-pku.cn). GSE65071 was used to acquire the miR-199b-3p expression data from the Gene Expression Omnibus (GEO) database (https://www.ncbi.nlm.nih.gov/geo/). The potential association between miR-199b-3p and CCDC88A was obtained from TargetScan (http://www.targetscan.org/vert 80/).

### Patients and specimens

A total of 49 patients were included, who underwent surgical treatment from 2010 to 2020 in our hospital. A total of 49 pairs of primary OS and adjacent non-cancerous bone tissues were obtained. All the tissues were collected and immediately cryopreserved at − 80 °C during the operation. The World Health Organization histological criteria and the American Joint Committee on Cancer (AJCC) staging system were used to confirm the diagnosis of OS. The AJCC staging system was recommended for the evaluation of the prognosis and the survival of patients with OS^20^.

### Inclusion and exclusion criteria

Inclusion criteria were as follows: osteosarcoma patients with an expected survival over 3 months without radiotherapy or chemotherapy before surgery. Exclusion criteria were as follows: (i) osteosarcoma patients who received radiotherapy or chemotherapy before surgery; and (ii) patients who failed to cooperate sign the informed consent form.

### Ethical statement

The proposal was reviewed and approved by the Ethics Committee of our hospital prior to implementation (October 2020; approval no. 2020119). Written informed consent was obtained from the guardians of the patients included in the present study.

### Cell culture

All cell lines, including hFOB, MG63 (RRID: CVCL_0426), and U2OS were purchased from Suzhou Culture Collection. All the cell lines were authenticated by STR analysis. DMEM (Invitrogen; Thermo Fisher Scientific, Inc.) containing 10% FBS (Gibco; Thermo Fisher Scientific, Inc.), streptomycin (100 μg/ml), and penicillin (100 U/ml) were used for cell culture. The cells were incubated in a humidified incubator containing 5% CO_2_ at 37 °C.

### Reverse transcription-quantitative PCR (RT-qPCR) assays

TRIzol (Invitrogen; Thermo Fisher Scientific, Inc.) was used to extract the total RNA from cells and tissues in accordance with the instructions provided by the kits. β-actin and U6 were used as the internal reference controls of CCDC88A and miR-199b-3p, respectively. The primers used are listed in Table 1. All experiments were performed in triplicate.

**Table 1:** The primer sequences used for the RT-qPCR analysis.

Gene	Primer sequence (5′–3′)
miR-199b-3p	F:AACACGCACAGTAGTCTGCA
	R: GTCGTATCCAGTGCAGGGT
CCDC88A	F: TGAAGAGCGGGATGGTCTCC
	R: TGCCAGTTCCACCGACAGAT
U6	F: CTCGCTTCGGCAGCACA
	R: AACGCTTCACGAATTTGCGT
β-Actin	F: TCACCCACACTGTGCCCATCTACGA
	R: CAGCGGAACCGCTCATTGCCAATGG

*RT-qPCR* reverse transcription-quantitative PCR, *miR* microRNA, *CCDC88A* coiled-coil domain containing 88A.

### Small interfering RNA (siRNA) transfection

miR-199b-3p mimics, miR-199b-3p inhibitor, siRNA targeting CCDC88A, and their corresponding control groups were purchased from Shanghai Genepharma Inc. The method of siRNA transfection was performed as described previously^21^. The sequences used are listed in Table 2. Each experiment was conducted in triplicate.

**Table 2:** The siRNA, mimic/inhibitor and negative control sequences.

Name	Sequence (5′–3′)
CCDC88A siRNA	AACGUUGGUUACACUACGUGA
Control CCDC88A siRNA	UUCUCCGAACGUGUCACGUAU
miR‑199b‑3p mimic	TGTCATCAGACGTGTAACCAAT
Control miR‑199b‑3p mimic	ACAGUAGUCUGCACAUUGGUUA
miR‑199b‑3p inhibitor	TAACCAATGTGCAGACTACTGT
Control miR‑199b‑3p inhibitor	UCACAACCUCCUAGAAAGAGUAGA

*siRNA* small interfering RNA, *miR* microRNA, *CCDC88A* coiled-coil domain containing 88A.

### Cell proliferation assay

Post-transfection, U2OS, and MG63 cells were seeded at a density of 1000 cells per well in 96-well plates. A total of 5 wells were used as replicates. A total of 10 μl 3-(4,5-dimethylthiazol-2-yl)-2,5-diphenyltetrazolium bromide (MTT) reagent (5 mg/ml) was incubated with the cells (0, 24, 48, and 72 h groups) at 37 °C for 2 h. Subsequently, 150 μl dimethyl sulfoxide was added to the wells. A microplate reader was used to measure the absorbance at a wavelength of 490 nm. All experiments were performed in triplicate.

### Transwell assay

The Transwell chamber was used to investigate OS cell migratory ability. A total of 200 μl cell suspension with only DMEM was plated into the upper chamber and 500 μl DMEM containing 20% FBS was added to the lower chamber. Subsequently, the cells were incubated for 48 h and the cells that did not penetrate through the membrane surface were removed with a cotton swab. The remaining cells were rinsed with PBS and paraformaldehyde was used to fix the cells for 10 min, followed by staining with 0.5% crystal violet. The number of cells that penetrated through the membrane was counted by an inverted microscope. Each experiment was conducted in triplicate.

### Luciferase reporter assay

MG63 and U2OS cells were seeded into 24-well plates. Subsequently, wild-type (wt) or mutant 3′-untranslated region (UTR) of CCDC88A, miR-199b-3p mimic/inhibitor, and negative control (NC) were transfected to the aforementioned cells using a Lipofectamine™ 2000 kit (Invitrogen; Thermo Fisher Scientific, Inc.). Following transfection, the cells were cultured for 48 h and the cell lysate was collected to measure luciferase activity using a dual-luciferase reporter gene assay system. The mimic/inhibitor and NC sequences used are listed in Table 2. Each experiment was conducted in triplicate (Supplementary Information).

### Western blot analysis

RIPA buffer (Biosharp Life Sciences) was used to extract the total proteins from MG63 and U2OS cells and the Bradford protein assay (Bio-Rad Laboratories, Inc.) was used to conduct protein quantitation. Electrophoresis was performed using 12% SDS-PAGE gels and the proteins were transferred to polyvinylidene fluoride membranes (EMD Millipore, Billerica, MA, USA). Subsequently, they were blocked with 5% fat-free milk at room temperature for 2 h. The membranes were cropped and incubated with primary antibodies at 4 °C overnight and with a secondary antibody for 1 h at room temperature. The bound antibodies were developed using enhanced chemiluminescence reagents (Pierce; Thermo Fisher Scientifc, Inc.). ImageJ software (version 1.42; National Institutes of Health) was used to measure the gray values. The antibodies used in the present study are shown in Table 3. Each experiment was conducted in triplicate.

**Table 3:** The list of the antibodies used for western blotting.

Antibody	Catalog number	Host	Dilution ratio	Company
β-Catenin	ab68183	Rabbit	1:1000	Abcam
c-Myc	ab32072	Rabbit	1:1000	Abcam
Cyclin D1	ab16663	Rabbit	1:1000	Abcam
Vimentin	ab92547	Rabbit	1:1000	Abcam
E-cadherin	ab40772	Rabbit	1:1000	Abcam
N-cadherin	ab76011	Rabbit	1:1000	Abcam
GAPDH	ab9485	Rabbit	1:1000	Abcam
Anti-rabbit lgG	ab150077	goat	1:4000	Abcam

### Statistical analysis

The data were analyzed by GraphPad Prism 8.0 (GraphPad Software, Inc.). The unpaired or paired Student t-tests were used for the determination of the differences in the quantitative data. The categorical variables were assessed by the χ^2^ or Fisher’s exact tests. The prognostic significance was determined by Kaplan–Meier and log-rank analyses. Receiver operating curve (ROC) and the area under the curve (AUC) were used for biomarker prediction. Pearson’s correlation was used to assess the strength of the linear correlation between two continuous variables. P < 0.05 was considered to indicate a significant difference.

### Ethics approval and consent to participate

The protocol was approved by the Ethics Committee of the Second People’s Hospital of Lianyungang (Lianyungang, China) in accordance with the guidelines of the ethics committee. And all experiments were performed in accordance with above relevant guidelines and regulations.

### Patient consent for publication

All patients and/or their legal guardians signed an informed consent form.

## Results

### The expression of CCDC88A is upregulated and miR-199b-3p expression is decreased in OS

**Figure 1:**
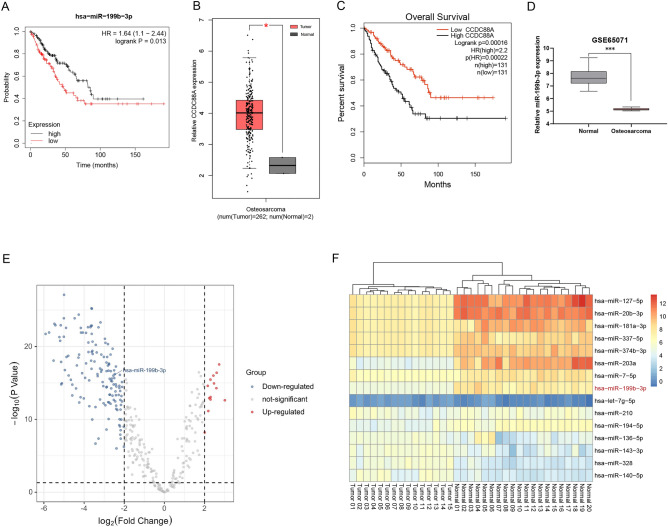
The expression levels of miR-199b-3p and CCDC88A are assessed in OS and are associated with disease prognosis as determined by bioinformatic analysis. (**A**) The patients with OS and high miR-199b-3p expression exhibited favorable survival (data from Kaplan–Meier plotter). (**B**) CCDC88A expression at the mRNA level was high in 262 tumor tissues and low in 2 normal tissues (data from GEPIA). (**C**) Patients with OS and high CCDC88A expression exhibited poor survival (data from GEPIA). (**D**) Based on GSE65071, the miR-199b-3p expression levels were dramatically decreased in OS samples. (**E**) Differentially expressed miRNAs were analyzed using a volcano plot between OS and normal samples (data from GSE65071). (**F**) The heat map depicts the partial differential miRNA expression in OS (data from GSE65071). *P < 0.05, ***P < 0.001. *miR* microRNA, *CCDC88A* coiled-coil domain containing 88A, *OS* osteosarcoma, *GEPIA* Gene Expression Profiling Interactive Analysis.

Elevated expression of miR-199b-3p was associated with the overall survival of patients with OS as determined by the Kaplan–Meier plotter (Fig. 1A). The relative expression of CCDC88A was found to be significantly higher in OS tissues following analysis of the GEPIA public database (Fig. 1B); it was also shown to be associated with poor overall survival (Fig. 1C). Based on GSE65071, the miR-199b-3p expression levels were dramatically decreased in OS samples (Fig. 1D). Differentially expressed miRNAs were analyzed using a volcano plot between OS and normal samples (Fig. 1E). The heat map depicts the partial differential miRNA expression in OS (Fig. 1F).

### Correlation of miR-199b-3p and CCDC88A expression levels with the clinical characteristics of patients with OS

**Figure 2:**
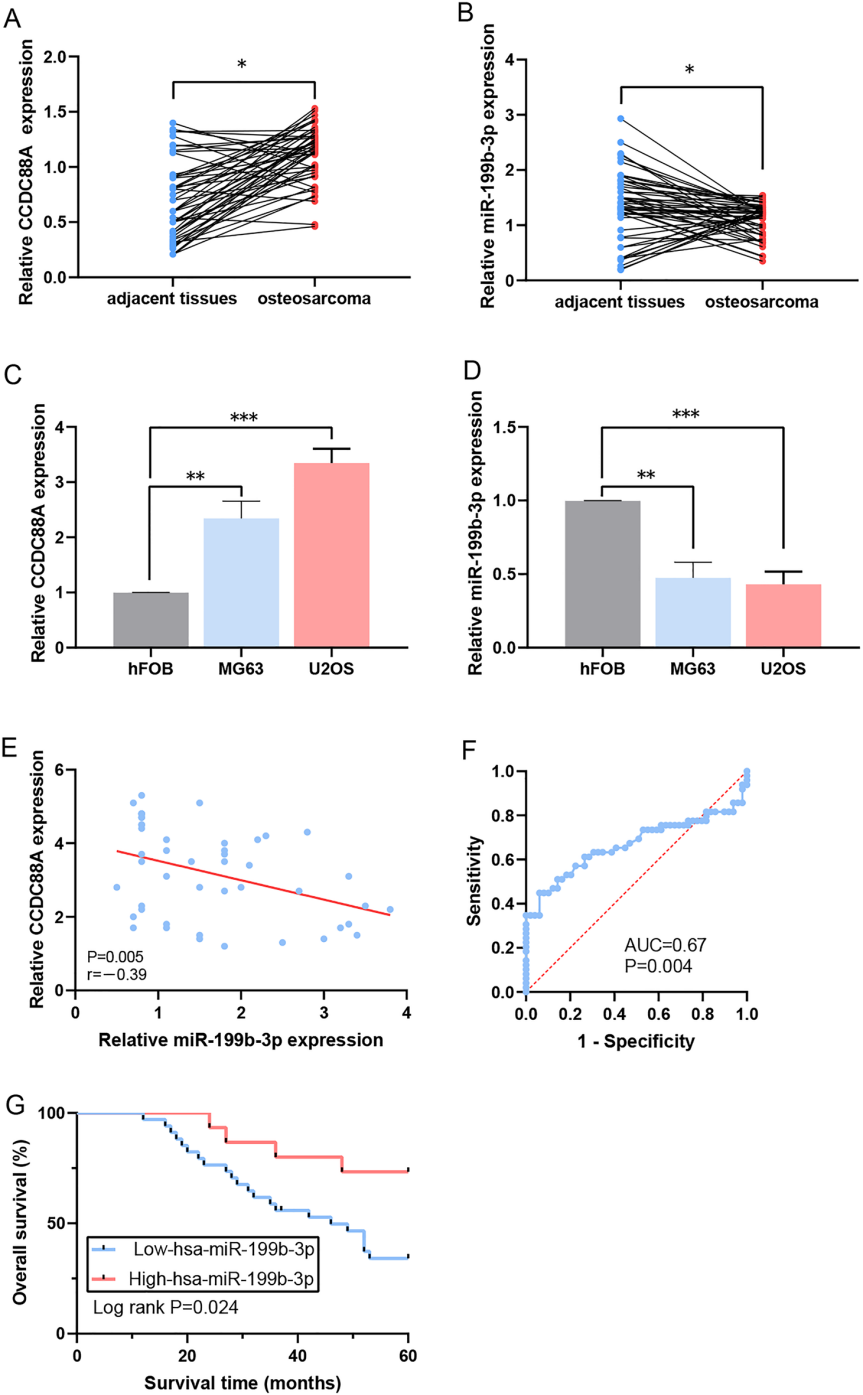
Determination of the expression levels of miR-199b-3p and CCDC88A in OS and association with disease prognosis. (**A**) The CCDC88A expression levels in the OS samples were higher than those in the normal samples, as determined by RT-qPCR analysis. (**B**) The miR-199b-3p expression levels in the OS samples were lower than those in the normal samples, as detected by RT-qPCR analysis. (**C**) The mRNA expression levels of CCDC88A were higher in OS cell lines compared with those noted in the human osteoblast cells, as determined by RT-qPCR analysis. (**D**) The expression levels of miR-199b-3p were lower in OS cell lines compared with those noted in the human osteoblast cells, as determined by RT-qPCR analysis. (**E**) Negative correlation between miR-199b-3p and CCDC88A expressions. (**F**) The cut-off value of miR-199b-3p was evaluated in patients with OS. (**G**) Determination of the survival curves for patients with OS with regard to miR-199b-3p expression. *P < 0.05, **P < 0.01, and ***P < 0.001. *miR* microRNA, *CCDC88A* coiled-coil domain containing 88A, *OS* osteosarcoma, *RT-qPCR* reverse transcription-quantitative polymerase chain reaction, *AUC* area under the curve. Each experiment was conducted in triplicate.

The expression levels of CCDC88A were higher in tumor tissues from clinical samples (Fig. 2A), whereas those of miR-199b-3p were lower (Fig. 2B). CCDC88A levels were increased in tumor cells compared with those of the normal osteoblast cell line hFOB1.19 (Fig. 2C). However, the expression levels of miR-199b-3p were decreased in OS cell lines (Fig. 2D). A negative correlation between CCDC88A and miR-199b-3p expressions was noted in patients with OS (P = 0.005, r = − 0.39; Fig. 2E). The best cut-off value was obtained by ROC analysis and two groups were selected, based on the expression levels of miR-199b-3p (P = 0.004, AUC = 0.67; Fig. 2F). Subsequently, the high expression group (n = 15) and low expression group (n = 34) were obtained (Table 4). Tumor size, distant metastasis, and clinical stage were found to be significantly associated with low expression levels of miR-199b-3p (P = 0.015, P = 0.047, and P = 0.029, respectively). Low miR-199b-3p expression levels were associated with a poor prognosis in patients with OS (P = 0.024) (Fig. 2G).

**Table 4:** Correlation of miR-199b-3p expression with the clinicopathological features of the patients with OS.

Clinicopathological features	Number of cases	miR-199b-3p expression		P-value
		High (n)	Low (n)	
Age (years)				0.323
< 8	16	3	13	
≥ 8	33	12	21	
Gender				0.753
Male	30	10	20	
Female	19	5	14	
Tumor size (cm)				0.015
< 8	26	12	14	
≥ 8	23	03	20	
Anatomic location				0.197
Tibia/femur	31	12	19	
Elsewhere	18	03	15	
Serum level of lactate dehydrogenase				0.352
Elevated	23	09	14	
Normal	26	06	20	
Serum level of alkaline phosphatase				0.356
Elevated	31	08	23	
Normal	18	07	11	
Clinical stage				0.047
I	16	08	08	
II	15	05	10	
III	18	02	16	
Distant metastasis				0.029
Absent	27	12	15	
Present	22	03	19	
Response to chemotherapy				0.345
Good	19	04	15	
Poor	30	11	19	

*miR* microRNA, *OS* osteosarcoma.

### miR-199b-3p inhibits OS cell proliferation and migration

**Figure 3:**
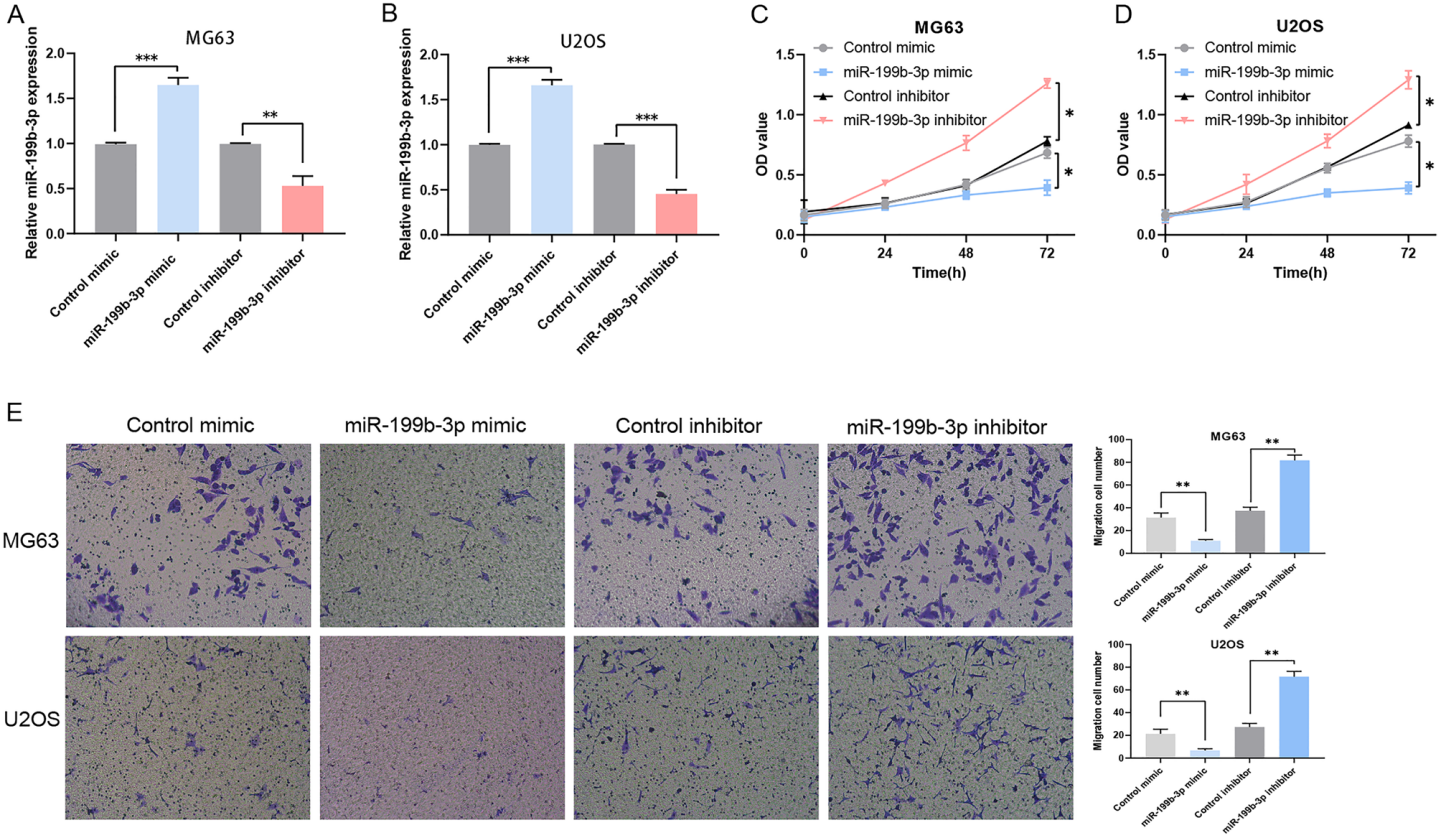
The proliferation and invasion of OS cells are suppressed by miR-199b-3p. (**A**) The expression levels of miR-199b-3p were measured by RT-qPCR in MG63 cells transfected with miR mimics and a miR inhibitor. (**B**) The expression levels of miR-199b-3p were measured by RT-qPCR in U2OS cells transfected with miR mimics and a miR inhibitor. (**C**) MTT analysis indicated the proliferative activity of MG63 cells transfected with miR-199b-3p mimics and a miR inhibitor. (**D**) MTT analysis indicated the proliferative activity of U2OS cells following transfection with miR-199b-3p mimics and a miR inhibitor. (**E**) The migration activity was measured following the increase and decrease in the expression levels of miR-199b-3p in MG63 and U2OS cells. *P < 0.05, **P < 0.01, and ***P < 0.001. *OS* osteosarcoma, *miR* microRNA, *RT-qPCR* reverse transcription-quantitative polymerase chain reaction, *MTT* 3-(4,5-dimethylthiazol-2-yl)-2,5-diphenyltetrazolium bromide, *OD* optical density. Each experiment was conducted in triplicate.

A miR-199b-3p mimic or inhibitor was transfected to MG63 and U2OS cells in order to induce upregulation or downregulation of miR-199b-3p expression. RT-qPCR was used to detect the efficiency of the transfection process (Fig. 3A,B). The results of the MTT assays indicated that the proliferation of the OS cell lines was significantly decreased following transfection of the cells with miR-199b-3p mimics, while that of the OS cell lines was promoted following transfection of the cells with miR-199b-3p inhibitors (Fig. 3C,D). Transwell assays indicated that the migratory activity levels of the OS cell lines were significantly decreased following transfection of the cells with miR-199b-3p mimics, while transfection of the cells with the miR-199b-3p inhibitors promoted the migratory activity of OS cells (Fig. 3E).

### miR-199b-3p targets CCDC88A and decreases CCDC88A expression in OS cell lines

**Figure 4:**
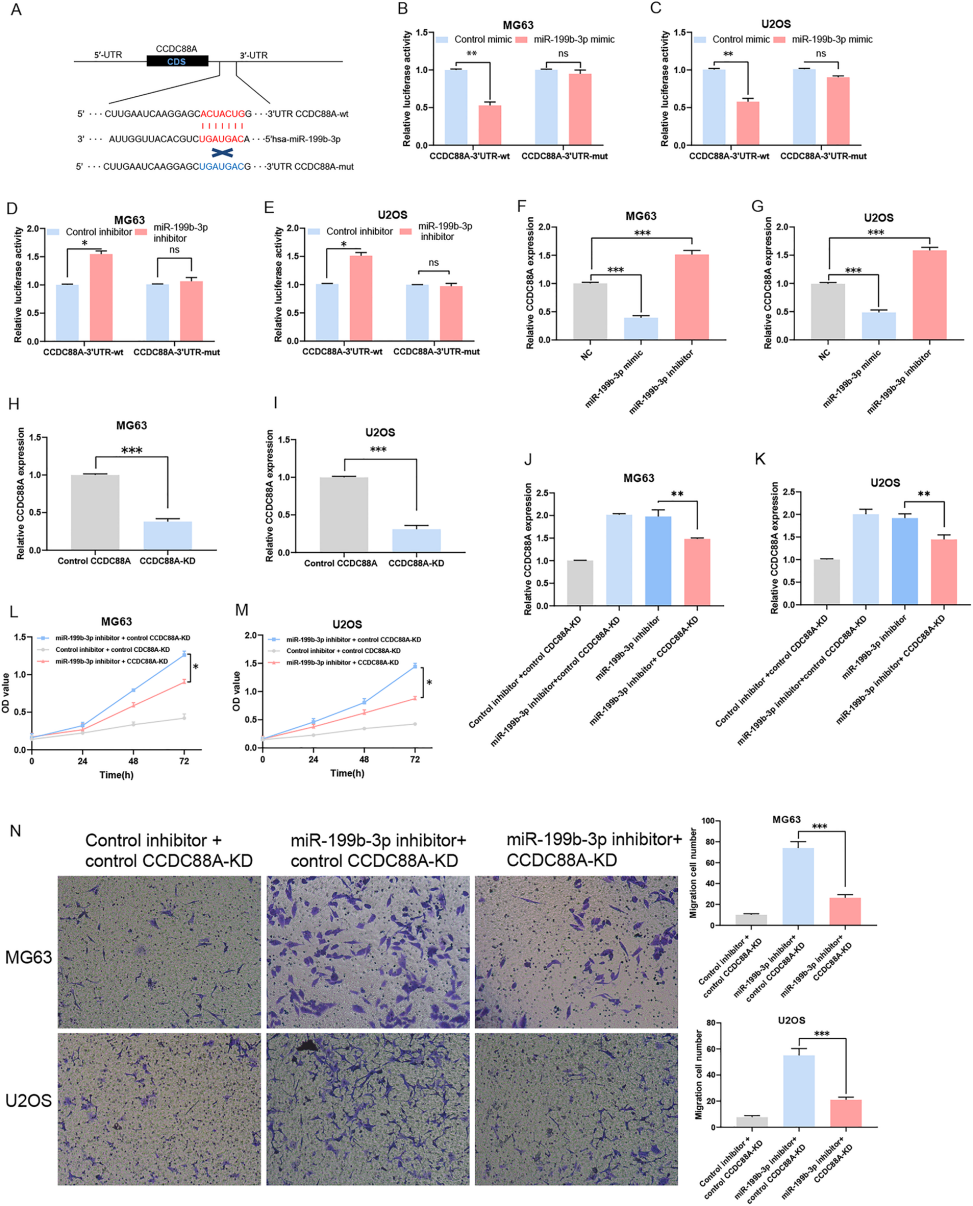
miR-199b-3p downregulates CCDC88A expression levels by direct binding to its 3′UTR. (**A**) The common target site of miR-199b-3p and CCDC88A 3′UTR. (**B**–**E**) Luciferase reporter gene assays. (**F**) Determination of the expression levels of CCDC88A in MG63 cells following transfection with miR-199b-3p mimics/inhibitors. (**G**) Determination of the expression levels of CCDC88A in U2OS cells following transfection with miR-199b-3p mimics/inhibitors. (**H**) The mRNA expression levels of CCDC88A were evaluated in MG63 cells co-transfected with CCDC88A siRNA by RT-qPCR. (**I**) The mRNA expression levels of CCDC88A in U2OS cells co-transfected with CCDC88A siRNA were detected by RT-qPCR. (**J**) The mRNA levels of CCDC88A in MG63 cells co-transfected with CCDC88A siRNA and the miR-199b-3p inhibitor were detected by RT-qPCR. (**K**) The mRNA levels of CCDC88A in U2OS cells co-transfected with CCDC88A siRNA and the miR-199b-3p inhibitor were detected by RT-qPCR. (**L**) The proliferative activity of MG63 cells co-transfected with CCDC88A siRNA and the miR-199b-3p inhibitor was detected by the MTT assay. (**M**) The proliferative activity of U2OS cells co-transfected with CCDC88A siRNA and the miR-199b-3p inhibitor was detected by the MTT assay. (**N**) The invasive activity of MG63 and U2OS cells co-transfected with CCDC88A siRNA and the miR-199b-3p inhibitor was determined by the Transwell assay. *P < 0.05, **P < 0.01, and ***P < 0.001. *miR* microRNA, *CCDC88A* coiled-coil domain containing 88A, *UTR* untranslated region, *siRNA* small interfering RNA, *RT-qPCR* reverse transcription-quantitative polymerase chain reaction, *MTT* 3-(4,5-dimethylthiazol-2-yl)-2,5-diphenyltetrazolium bromide, *KD* knockdown, *NC* negative control, *OD* optical density, *wt* wild-type, *mut* mutant, *ns* not significant, *CDS* coding sequence. Each experiment was conducted in triplicate.

Previous experiments revealed that the CCDC88A 3′UTR and miR-199b-3p had a putative binding site as determined by the TargetScan database (Fig. 4A). Luciferase reporter gene assay demonstrated in the CCDC88A-wt group that the luciferase activity was significantly inhibited by miR-199b-3p mimics (Fig. 4B,C), while in the CCDC88A-wt group, the luciferase activity was promoted by miR-199b-3p inhibition (Fig. 4D,E). Subsequently, CCDC88A levels were measured by RT-qPCR to determine if miR-199b-3p could alter CCDC88A expression. In addition, the expression levels of CCDC88A were decreased in the miR-199b-3p mimics group and increased in the miR-199b-3p inhibitor group compared with those of the NC group (Fig. 4F,G). Therefore, it was concluded that miR-199b-3p had a significant influence on OS by targeting CCDC88A.

### CCDC88A knockdown reverses the miR-199b-3p-mediated capacity of proliferation and invasion

The knockdown efficiency of CCDC88A was explored by RT-qPCR analysis (Fig. 4H,I). It was found that CCDC88A expression was increased following transfection with miR-199b-3p inhibitors in MG63 and U2OS cells compared with those of the control group; however, it was inversely increased when cotransfected with miR-199b-3p inhibitors (Fig. 4J,K). The results of the MTT assays revealed that the proliferation of OS cells could be reversed by knockdown of CCDC88A expression (Fig. 4L,M). The results of the Transwell assay indicated that the invasion of OS cells could be reversed by knockdown of CCDC88A expression (Fig. 4N).

### The miR-199b-3p-CCDC88A axis regulates the malignant behavior of OS cells via the EMT and Wnt/beta-catenin signaling pathways

**Figure 5:**
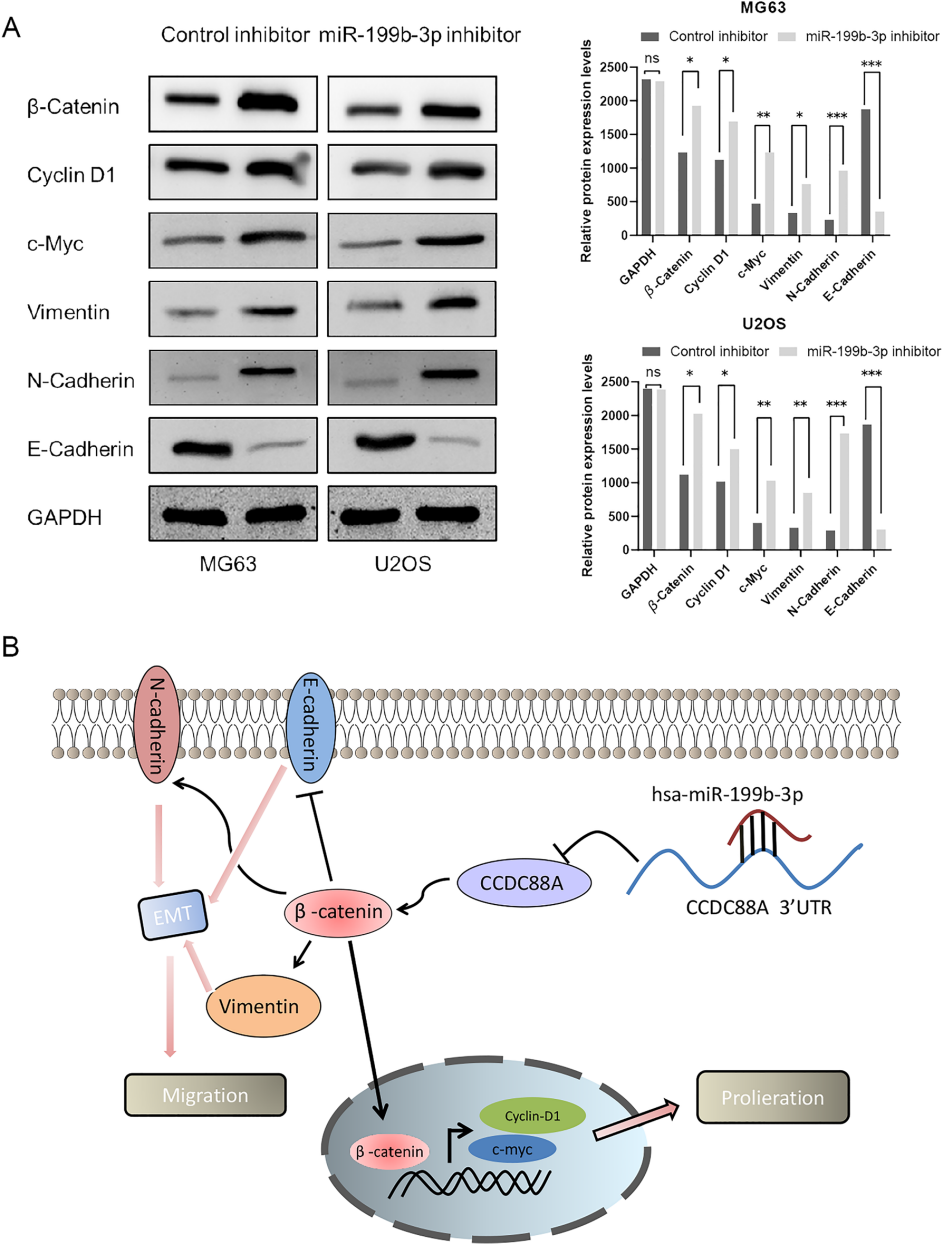
The miR-199b-3p/CCDC88A axis regulates the OS cell malignant behaviors via the Wnt/β-catenin pathway and the EMT process in vitro. (**A**) Inhibition of the expression of miR-199b-3p activates the Wnt/β-catenin signaling pathway and the epithelial–mesenchymal transition process. (**B**) The miR-199b-3p/CCDC88A axis regulates OS cell malignant behavior via the Wnt/β-catenin pathway and the EMT process. *P < 0.05, **P < 0.01, and ***P < 0.001. *miR* microRNA, *CCDC88A* coiled-coil domain containing 88A, *OS* osteosarcoma, *EMT* epithelial-to-mesenchymal transition, *UTR* untranslated region, *EMT* epithelial–mesenchymal transition. Each experiment was conducted in triplicate. Note: during development the western blotting members, due to exposure and contrast adjustment, the edges of some members were not clearly displayed in pictures.

The protein levels of β-catenin, E-cadherin, c-myc, cyclin D1, vimentin, and N-cadherin were detected in OS cells overexpressing miR-199b-3p. It was shown that when miR-199b-3p expression was suppressed in OS cells, the expression levels of all of the aforementioned key proteins were increased except for E-cadherin (Fig. 5A). Taken together, the results indicated that CCDC88A regulated by miR-199b-3p induced tumor proliferation and invasion in OS via the EMT and Wnt/beta-catenin signaling pathways (Fig. 5B).

## Discussion

As a novel regulator, miR-199b-3p, has particular relevance to tumorigenesis in a wide range of tumors^14,15^. In addition, overexpression of miR-199b-3p hindered the proliferation of colorectal cancer cells and induced their apoptosis by decreasing cysteine-rich motor neuron 1 (CRIM1) via the Wnt/beta-catenin pathway^15^. In prostate cancer, it has been reported that miR-199b-3p can target phospholipase c epsilon and consequently suppress malignant proliferation^8^. It was also shown that lower miR-199b-3p expression correlated with poor prognosis of patients with prostate cancer^14^.

In the present study, GSE65071 was analyzed from the GEO database and the data demonstrated that the expression levels of miR-199b-3p were downregulated in OS. This finding was subsequently verified in OS cell lines. In the 49 cases with OS, the expression levels of miR-199b-3p were also significantly lower in cancer tissues compared with those noted in paracancerous tissues. Based on the patient tissue data, ROC curve analysis was used to obtain the value of AUC, which was estimated to be 0.67, indicating that miR-199b-3p was a sensitive diagnostic predictor of OS. In order to investigate the biological functions of miR-199b-3p, MTT and Transwell assays were implemented. It was revealed that the proliferation and invasion were inhibited following the upregulation of miR-199b-3p in MG63 and U2OS cell lines. According to the Kaplan–Meier analysis, lower miR-199b-3p expression was associated with a shorter overall survival time. Previous studies have demonstrated that miR-199b-3p exerts its function as a tumor suppressor in prostate cancer, colorectal carcinoma, and bladder cancer whereas it acts as an oncogene in pancreatic cancer^14,15,22^. Based on this evidence, the present study investigated the potential target of miR-199b-3p in OS.

In the present study, miR-199b-3p was found to possess a potential binding site with CCDC88A by TargetScan. CCDC88A has been reported to play an important role in tumor progression and it has also been confirmed to function as an oncogene in tumors^23^. Based on these studies, it is deduced that CCDC88A is involved in tumor progression of several cancer types, such as breast, colon, and cervical cancer^24–26^. It also has been reported that CCDC88A is involved in actin cytoskeleton formation and enhances Akt phosphorylation, and it also acts downstream of the PI3K/Akt signalling pathway and is directly activated by Akt^27^. CCDC88A also be reported can bind to and activate Gαi3, which further activates the PI3K/Akt signalling pathway^28^. A researcher about glioblastoma have also confirmed that CCDC88A expression is closely related to tumour malignancy, including the histological grade and metastasis, as well as progression-free survival and overall survival^29^. In human colorectal cancer, CCDC88A was associated with TNM tumor stage and the rates of liver metastasis and other distant metastases^30^. However, the effect of CCDC88A on OS progression and its regulatory mechanism remain unclear. To date, CCDC88A has not been studied in OS. In our study the relative expression of CCDC88A was found to be significantly higher in OS tissues and it was also shown to be associated with poor overall survival. The link between CCDC88A and miR-199b-3p was attested by luciferase reporter assays in the present study. More importantly, it was observed the CCDC88A expression levels were decreased in OS cell lines with upregulated miR-199b-3p levels. This demonstrated that miR-199b-3p and CCDC88A had a negative association, which was consistent with the findings in the clinical specimens. Strikingly, it was found that CCDC88A could partially reverse the inhibitory effect caused by the induction of miR-199b-3p. The present study demonstrated that miR-199b-3p could bind to CCDC88A and reverse its inhibitory effect on the proliferation and invasion of the OS cell lines.

Subsequently, the molecular mechanism of the regulation of OS aggressiveness by the miR-199b-3p/CCDC88A axis was also investigated. It has been reported that miR-199b-3p may modulate proliferation and invasion in colorectal cancer via the Wnt/beta-catenin signaling pathway by targetting CRIM1^15^. It has also been shown that the Wnt/beta-catenin pathway can facilitate cancer cell proliferation and differentiation, which play a fundamental role in tumorigenesis^31^. In the present study, the data indicated that miR-199b-3p may inhibit OS cell proliferation via the Wnt/β-catenin pathway. These findings are consistent with the results reported in a previous study. EMT is considered to be an embryonic process due to decreased cell–cell adherence complexes. It endows cells with enhanced migratory and invasive properties and promotes tumor metastasis^32^. Cancer cells may exhibit EMT alterations due to low expression levels of E-cadherin and high expression levels of N-cadherin and vimentin^33^. In 2021, it was reported that miR-199b-3p could inhibit EMT and cause dysfunction of the renal tubules in diabetic nephropathy^34^. In the present study, it was found that miR-199b-3p may affect cell invasion via EMT in OS.

Overall, the present study demonstrated for the first time that the miR-199b-3p/CCDC88A axis regulated OS development via the EMT process and the Wnt/beta-catenin pathway. However, the present study contains certain limitations. It was a retrospective study, which was conducted in vitro*,* may not reflect the behavior of OS in vivo; and only 49 cases were included, the sample size was small and generalizability may be limited. Therefore, future studies should address these limitations.

In conclusion, the results of the present study indicated that the miR-199b-3p/CCDC88A axis could play an important role in OS progression by affecting the EMT and the Wnt/beta-catenin signaling pathways.

## Supplementary Information

Supplementary Information.

## Data Availability

The datasets generated in the present study may be requested from the corresponding author.
