# Supplementary figures and images for: hsa-miR-199b-3p suppresses osteosarcoma progression by targeting CCDC88A, inhibiting epithelial-to-mesenchymal transition, and Wnt/beta-catenin signaling pathway
Source: Sci Rep. 2023 Aug 2;13:12544. doi: 10.1038/s41598-023-39537-0 (PMC10397339; doi:10.1038/s41598-023-39537-0)

β-catenin

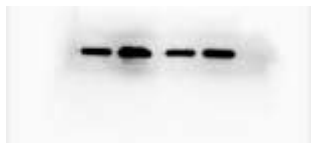

cyclin D1

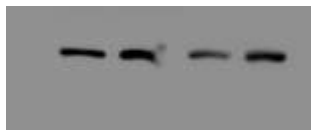

c-Myc

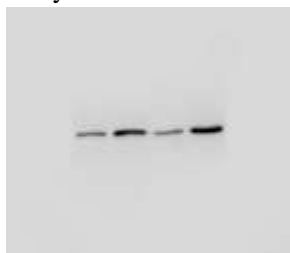

Vimentin

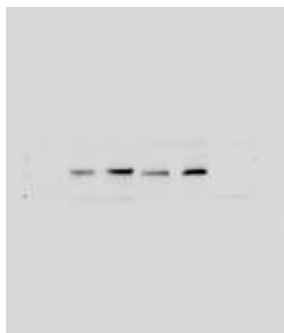

N-Cadherin

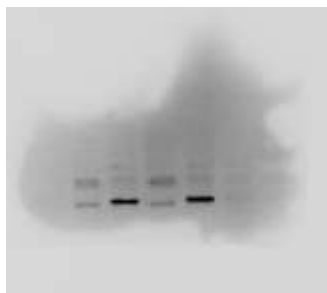

E-Cadherin

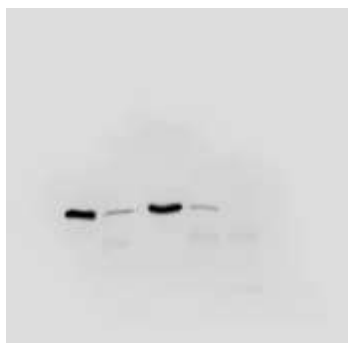

GAPDH

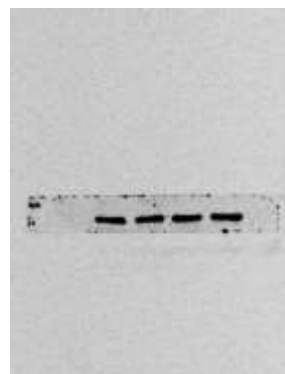

Supplement: Supplementary file 1 — Supplementary Information. [file 41598_2023_39537_MOESM1_ESM.pdf]
